# Early-Onset Depression Is Associated With Specific Neurovegetative Symptoms

**DOI:** 10.1192/j.eurpsy.2022.392

**Published:** 2022-09-01

**Authors:** E. Kasyanov, G. Rukavishnikov, A. Nikolishin, A. Gareeva, K. Savitskaya, A. Kazantseva, A. Kretsu, V. Soldatkin, T. Lezheyko, V. Golimbet, N. Neznanov, A. Kibitov, G. Mazo

**Affiliations:** 1 Bekhterev National Medical Center for Psychiatry and Neurology, Translational Psychiatry, Saint-Petersburg, Russian Federation; 2 Federal State Budgetary Istitution Serbsky National Medical Research Centre for Psychiatry and Narcology of Ministry of Heath of the Russian Federation, Laboratory Of Molecular Genetics, Moscow, Russian Federation; 3 Institute of Biochemistry and Genetics of Ufa Federal Research Center of the Russian Academy of Sciences, Laboratory Of Human Molecular Genetics, Ufa, Russian Federation; 4 Peoples’ Friendship University of Russia, Psychiatry, Moscow, Russian Federation; 5 Laboratory of Human Molecular Genetics, Institute Of Biochemistry And Genetics, Ufa, Russian Federation; 6 Saint-Petersburg State University N.I.Pirogov Clinic of High Medical Technologies, Tharpy, Saint Petersburg, Russian Federation; 7 Rostov State Medical University, Department Of Psychiatry, Addictology And Medical Psychology, Rostov-on-Don, Russian Federation; 8 Mental Health Research Center, Clinical Genetics Laboratory, Moscow, Russian Federation; 9 Bekhterev National Medical Center for Psychiatry and Neurology, Geriatric Psychiatry, Saint-Petersburg, Russian Federation; 10 Serbsky National Medical Research Center on Psychiatry and Addictions, Molecular Genetics Laboratory, Moscow, Russian Federation

**Keywords:** Depression, Family history, age at onset, Neurovegetative Symptoms

## Abstract

**Introduction:**

The age at onset of depression is not only an important clinical predictor of the further disease course, but also a robust marker, reflecting the genetic impact on depression risk.

**Objectives:**

This study aimed to find whether early-onset depression had an association with specific clinical symptoms, comorbid psychiatric disorders and family history of mood disorders.

**Methods:**

This pilot cross-sectional, multicenter study was performed under the supervision of the Russian National Consortium for Psychiatric Genetics. Early-onset depression was defined as the first depressive episode before the median age of onset in the sample (Me=29 years). Logistic regression models were used to determine the independent association of early-onset depression, after adjusting for the effects of sex and age, with binary characteristics.

**Results:**

A total of 172 patients with depression were enrolled in the study (64.5% women; age - 40.9 (15.9) years). Early-onset depression was associated with psychomotor retardation (p=0,025; OR=2,3; 95%CI [1,1 - 4,9]), decreased libido (p=0,014; OR=2,8; 95%CI [1,2 - 6,2]), and lower prevalence of weight loss/decreased appetite (p=0,011; OR=0,4; 95%CI [0,2 - 0,8]). No associations were found with the history of comorbid psychiatric disorders and the family history of mood disorders.

**Conclusions:**

Early-onset depression is associated with specific neurovegetative symptoms. Further clinical and genetic studies are needed to evaluate the specific effects of age at onset of depression on its clinical course.

**Disclosure:**

Research is supported by an RSF grant №20-15-00132.

